# Endemic Human Coronavirus-Specific Nasal Immunoglobulin A and Serum Immunoglobulin G Dynamics in Lower Respiratory Tract Infections

**DOI:** 10.3390/vaccines12010090

**Published:** 2024-01-17

**Authors:** Ferdyansyah Sechan, Katherine Loens, Herman Goossens, Margareta Ieven, Lia van der Hoek

**Affiliations:** 1 Laboratory of Experimental Virology, Department of Medical Microbiology and Infection Prevention, Amsterdam University Medical Centers, University of Amsterdam, Meibergdreef 9, 1105 AZ Amsterdam, The Netherlands; m.f.sechan@amsterdamumc.nl; 2 Amsterdam Institute for Infection and Immunity, 1105 AZ Amsterdam, The Netherlands; 3 Laboratory of Medical Microbiology, Vaccine & Infectious Disease Institute (VAXINFECTIO), University of Antwerp, 2610 Antwerp, Belgium; katherine.loens@uantwerpen.be (K.L.); herman.goossens@uza.be (H.G.); greet.ieven@uza.be (M.I.)

**Keywords:** HCoV-HKU1, HCoV-NL63, HCoV-229E, HCoV-OC43, immunoglobulin A, spike antibodies, nucleocapsid antibodies, mucosal immunity

## Abstract

Endemic human coronaviruses (HCoV) NL63, 229E, OC43, and HKU1 cause respiratory infection. Following infection, a virus-specific serum antibody rise is usually observed, coinciding with recovery. In some cases, an infection is not accompanied by an immunoglobulin G (IgG) antibody rise in serum in the first month after HCoV infection, even though the infection has cleared in that month and the patient has recovered. We investigated the possible role of nasal immunoglobulin A (IgA). We measured spike (S) and nucleocapsid (N)-specific nasal IgA during and after an HCoV lower respiratory tract infection (LRTI) and compared the IgA responses between subjects with and without a significant IgG rise in serum (IgG responders (*n* = 31) and IgG non-responders (*n* = 14)). We found that most IgG responders also exhibited significant nasal IgA rise in the first month after the infection, whereas such an IgA rise was lacking in most IgG non-responders. Interestingly, the serum IgG non-responders presented with a significantly higher nasal IgA when they entered this study than during the acute phase of the LRTI. Our data suggest that nasal IgA could be part of a fast acute response to endemic HCoV infection and may play a role in clearing the infection.

## 1. Introduction

Endemic—also called seasonal—human coronaviruses (HCoVs) consist of four HCoV species: HCoV-NL63, HCoV-229E, HCoV-OC43, and HCoV-HKU1. They generally cause mild respiratory diseases, like common colds [1,2,3]. Following infection, a rise in serum antibodies against viral antigens, including spike (S) and nucleocapsid (N), is detected [4,5]. Recent studies from our research group further solidified this knowledge, with temporary rises of serum immunoglobulin G (IgG) against HCoV antigens throughout life [6,7]. However, in some acute LRTIs, we did not always observe a significant serum IgG rise against either the HCoV-S or -N antigen in the first month following the acute PCR-confirmed HCoV infection [7]. 

Virus-specific immunoglobulin A (IgA) in the mucosal layer is part of local immunity, which is raised against a viral infection at the site of infection [8]. This antibody subset neutralizes viruses, toxins, and bacteria in the mucosal layer to prevent infection and colonization [9,10]. For example, mucosal IgA production—as a result of oral live-attenuated virus vaccination—is recognized as a factor of poliovirus clearance in the gastrointestinal tract of infants [11,12]. Nasal vaccination using inactivated whole particles of influenza A virus (IAV) results in IgA production with strong virus neutralization activity [13,14]. High levels of respiratory syncytial virus (RSV)-specific nasal IgA also correlate with protection against experimental RSV infection [15], and the same was found for HCoV-229E, with subjects that carry high pre-infection IgA concentrations remaining uninfected after experimental nasal inoculation with an HCoV-229E virus culture [4,16]. Similarly, for SARS-CoV-2, virus-specific nasal IgA level was inversely correlated with viral load [17,18,19], and higher nasal IgA level was associated with lower odds for SARS-CoV-2 infection [20]. We, therefore, hypothesize that an absence of serum IgG rise in response to acute HCoV infection could be explained by IgA clearing the infection in the nasal mucosa. The IgA-mediated clearance may eliminate the trigger for IgG production. 

A local immune reaction can be recognized by either a high mucosal IgA concentration at the acute phase of the disease or a mucosal IgA increase after recovery, which can both be measured in nasopharyngeal swab (NPS) samples. In our study, we compared the levels of IgA targeting the nucleocapsid (N) and spike (S) antigens and the rise in IgA targeting these HCoV proteins in the first month of an LRTI caused by an HCoV infection. We specifically focused on people with or without a significant IgG rise in serum. 

## 2. Materials and Methods

### 2.1. The GRACE Study

The Genomics to Combat Resistance against Antibiotics in Community-acquired LRTI in Europe (GRACE) study investigated the viral and bacterial etiology of community-acquired lower respiratory tract infection (LRTI). Details of this study are described elsewhere [21]. Briefly, study participants were recruited between November 2007 and April 2010 by primary care practitioners in 16 networks from 12 European countries. Participation was voluntary and without monetary reward. Written informed consent was obtained before enrollment. Each subject was invited to join this study when they sought care from their general practitioner (GP) for symptoms that could indicate acute (three weeks or fewer) lower respiratory tract infection. All recruiting GPs received standardized sampling material and a protocol with detailed instructions on the sampling and data collection of the patients. The NPS and blood of each subject were first sampled within 24 h of study enrollment and inclusion (Visit 1 (V1)), and sampling was repeated roughly 28 days later (Visit 2 (V2)). Demographic data and clinical manifestation of LRTI for each subject were also recorded by the GPs during V1 using a standardized case report form (CRF). The data collected by the CRF included age, gender, presence of comorbidities, estimated date when cough or other symptom was experienced before the first visit, and the presence and severity of 14 LRTI-related symptoms, such as cough, phlegm, fever, and runny nose. Serum and NPS samples in universal transport medium (UTM, Copan, Brescia, Italy) were stored frozen in the local laboratories until regular shipment to the central laboratory (University Hospital Antwerp), where specimens were stored at −80 °C until analysis. Etiological agents causing LRTI were measured as described [22]. For HCoVs, in-house PCR tests for HCoV-229E, HCoV-OC43, and HCoV-NL63 [22] or commercial RT-PCR multiplex assays for HCoV-HKU1 (RespiFinder Plus, PathoFinder [22]) were performed on the NPS samples. 

We have previously studied GRACE study subjects (NL-01 to NL-11, TT-01 to TT-13, OC-01 to OC-14, and HK-01 to HK-13). These people were infected (PCR-confirmed) by one of the endemic HCoVs at V1, yet they were PCR-negative for the virus at V2. Eighty-two percent of the V1-NPS were sampled in the first week of illness. The HCoV-Ct values at V1, which were available for 34 of the 45 LRTIs, showed no significant correlation with the duration of illness prior to GP visit (ρ = −0.09, *p* = 0.615. Figure S1A, left panel) or the duration of cough prior to GP visit at V1 (r = −0.01, *p* = 0.954, Figure S1A, right panel) or the nasal IgA level at V1 (ρ = 0.27, *p* = 0.129, Figure S1B).

### 2.2. Threshold for Serum IgG Responder/Non-Responder Categorization 

The cut-off to determine a significant antibody response (or a non-response) for serum IgG was based on the anti-N ELISA test. We previously monitored serum IgG dynamics after acute HCoV infection [7]. Antibody dynamic was defined as the fold change of ELISA signal at V2 compared to V1, and a significant antibody increase resulting from viral infection is defined as an anti-N fold-change value of ≥1.4, as described in detail by Edridge et al. [6]. In short, Edridge et al. showed the natural fluctuation in antibodies determined for measles virus antibodies. Fold changes in antibody ELISA signals for measles virus ranged between 0.85 and 1.28. A threshold for coronavirus infection was subsequently determined by evaluating the distribution of the HCoV anti-N ELISA signal fold change during 2473 months of follow-up in ten individuals with more than 15 years of follow-up and serum sampling every 6 months. During most intervals, no coronavirus infection occurred, and infections thus appeared as outlier fold changes. Outliers were found for signal fold changes ≥ 1.40, and these values were indeed accompanied by self-reported influenza-like illnesses [6]. In contrast, the anti-S IgG was not used to distinguish serum IgG responders and non-responders. Anti-S was measured via Luminex assay (see below for assay details). For this test, no longitudinal analysis of natural variation in serum, as described above, has been done; therefore, we only used the anti-N fold changes in the current study to define serum IgG responders and non-responders.

### 2.3. Study Participants

For 45 of the 54 study participants, the collection of V1 clinical material was sufficiently close to the start of the illness (median and interquartile range (IQR) is 4 (3–6.25) days, 16 days max), and the nasopharyngeal swab material collected at both V1 and V2 were available for an IgA study. Based on their serum anti-N IgG fold rise (>1.4), 31 of the 45 were grouped as serum IgG responders (HCoV-NL63 *n* = 9, HCoV-229E *n* = 11, HCoV-OC43 *n* = 8, and HCoV-HKU1 *n* = 3) [7]. The rest, *n* = 14 (HCoV-229E *n* = 1, HCoV-OC43 *n* = 5, and HCoV-HKU1 *n* = 8), were grouped as serum IgG non-responders. 

### 2.4. Multiplex Anti-HCoV IgG and IgA Assay 

The serum anti-S IgG was measured using an in-house multiplex assay (Luminex, Austin, TX, USA) as previously described [7]. The nasal anti-N IgA and nasal anti-S IgA assays were conducted similarly, with some modifications, namely nasopharyngeal swab material suspended in transport medium (NPS) was diluted 1:20 in blocking buffer. Goat-anti-human IgA-PE (Southern Biotech, Birmingham, AL, USA) was used as the secondary antibody, and uncoupled beads were included as negative control. Antibody concentrations were expressed as median fluorescence intensity (MFI) of at least 50 beads per antigen. Only the assay signal value of the antigen that matched the infecting HCoV species, as determined by the PCR assay, was considered for further analysis. Similar to the serum HCoV anti-N IgG dynamics, we expressed anti-HCoV antibody dynamics as fold-change values (the ratio of V2:V1 antibody levels). 

### 2.5. Data Analysis

We compared both the fold-change values of nasal IgA, as well as the actual IgA levels at V1 and V2, between the serum IgG responders and serum IgG non-responders. The distribution of continuous variables (antibody values and fold changes, age of participants, and days of illness prior to sampling) was compared with Mann–Whitney test, and categorical variables (sex, smoking history, comorbidities, and symptoms) using 2-tailed Fisher exact test. Correlation between two continuous variables was evaluated using Spearman’s rank correlation test. All statistical analyses were done with Prism software version 9.5.1 (GraphPad). 

## 3. Results

**Figure 1:**
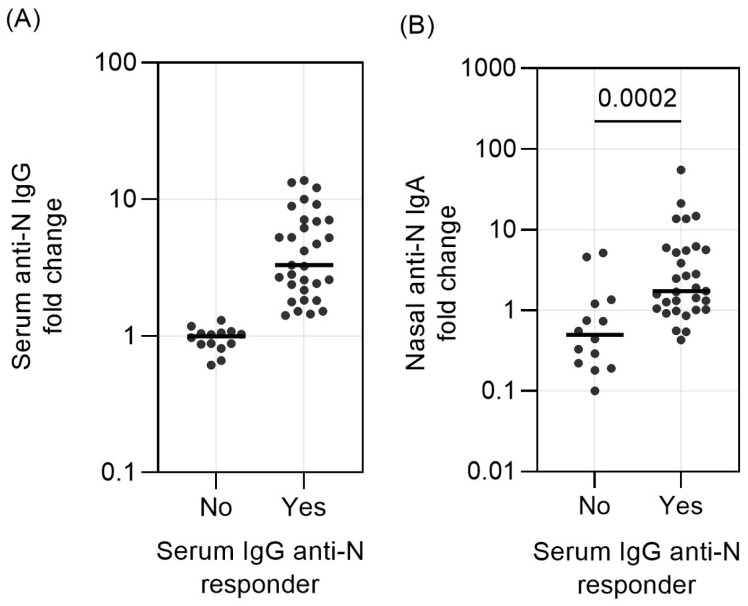
Changes of serum anti-N IgG and nasal anti-N IgA in the serum IgG responders and serum IgG non-responders. (**A**) The fold-change value of serum anti-N IgG between responders and non-responders. (**B**) The fold-change value of nasal anti-N IgA between responders and non-responders. Each dot represents one subject, and median of each dataset is denoted as a horizontal solid bar. Value distribution between groups was compared with Mann –Whitney test, and significance is defined as *p* < 0.05.

We enrolled 45 subjects with an LRTI from the GRACE study, infected by a single endemic HCoV species (HCoV-NL63 *n* = 9, HCoV-229E *n* = 12, HCoV-OC43 *n* = 13, and HCoV-HKU1 *n* = 11). These subjects were divided into serum IgG responders (*n* = 31) and serum IgG non-responders (*n* = 14) based on the serum anti-N IgG rise specific to the virus (Figure 1A, Table S1). The distribution of sex, age, past/current smoking history, comorbidities, ILI symptoms, and days of illness prior to the first visit (V1) to the general practitioner was also similar for the two groups (Table 1).

We subsequently tested for nasal anti-N IgA and calculated the fold-change values (V2 vs. V1). In the group of serum IgG responders (fold change 1.4 or higher), the nasal IgA fold change was also above 1 (median value of 1.73 (Figure 1B)). In contrast, at least half of the fold-change values of the non-responders were below 1, with a median value of 0.50. Thus, the nasal anti-N IgA fold-change values were significantly different between serum IgG responders and serum IgG non-responders (*p* = 0.0002, Figure 1B, Table S2).

**Figure 2:**
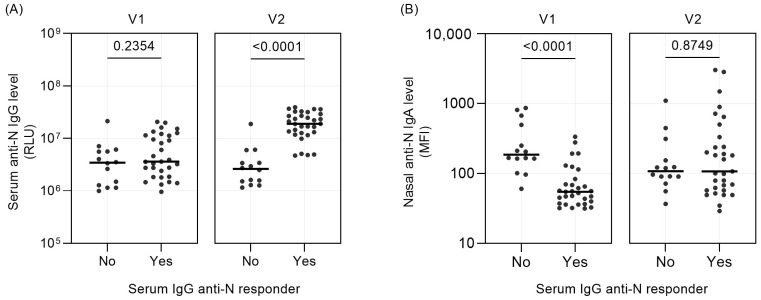
Anti-N antibody values at V1 and V2 between responders and non-responders. (**A**) Serum anti-N IgG level in relative luminescence unit (RLU) at V1 and V2, grouped between responders and non-responders. (**B**) Nasal anti-N IgA level in median fluorescence unit (MFI) at V1 (left panel) and V2 (right panel), grouped between responders and non-responders. Each dot represents one subject, and median of each dataset is denoted as a horizontal solid bar. Value distribution between groups was compared with Mann–Whitney test, and significance is defined as *p* < 0.05.

The finding that the majority of serum IgG non-responders have a nasal IgA fold change of 1 or less was unexpected. This shows that the IgA concentration was actually decreasing following the infection instead of rising. We theorized that the IgA response of the IgG non-responders may actually have been very fast and that the first NPS sample may have been collected during the peak of local IgA production. To test this assumption, we compared the nasal IgA antibody values at V1 and V2. Indeed, the nasal anti-N IgA differed significantly at V1 (*p* < 0.0001), with the IgG non-responder group showing higher values of mucosal IgA at V1 (Figure 2B, left panel; Table S2). 

The comparisons between the responders and the non-responders mentioned above were all done for antibodies recognizing the N-protein of the viruses. We also measured the levels and the dynamics of antibodies targeting the S protein of the virus on the serum and the NPS. Anti-S IgG in serum and anti-S IgA in nasal samples were examined in a similar manner as the anti-N antibodies (Table S3). Four of the anti-N IgG serum non-responders (OC-05, OC-13, HK-04, and HK-08) showed a substantial fold rise (fold-change values of 42.03, 514.42, 261.57, and 18.82, respectively). Concerning the nasal anti-S IgA fold change, the group of serum IgG responders showed a median fold rise of 4.56 (IQR: 1.45–18.26), while the median fold change for nasal spike IgA was around 1 (IQR: 0.13–39.48) for the group of serum IgG non-responders (Table S4, Figure S2A). Similar to our findings for anti-N nasal IgA, the nasal anti-spike IgA concentration at V1 was significantly higher in serum IgG non-responders (*p* = 0.042, Table S4, Figure S2B, left panel). Of note, if we would categorize the serum anti-N IgG non-responders with high serum anti-S IgG rise (OC-05, OC-13, HK-04, and HK-08) as “Responders”, the link between high nasal IgA recognizing the N or S antigen at V1 and being non-responders is still significant (*p* < 0.0001 and *p* = 0.012 for anti-N and anti-S, respectively).

We checked whether the level of nasal anti-N antibodies at V1 was possibly influenced by the duration between the study enrollment and the date when the illness (or cough) first appeared. This date was retroactively recalled by the subjects as a part of the CRF data collected by the GPs. We, indeed, found some correlation between the duration of prior illness (days) and the value of nasal anti-N IgA (ρ = 0.30, *p* = 0.044, Figure S3A, left panel), and this trend was also noted when we looked at the duration of cough prior to V1 (ρ = 0.282, *p* = 0.061, Figure S3A, right panel). Besides that, we also found that the nasal anti-S IgA value at V1 of all subjects correlated with duration of illness (ρ = 0.31, *p* = 0.041) or cough (ρ = 0.35, *p* = 0.019) prior to enrollment (Figure S3B, left and right panel). 

Finally, we looked at the possible correlation between nasal anti-N IgA and nasal anti-S IgA. We found that the nasal anti-S IgA fold change was significantly correlated with nasal anti-N IgA fold change (ρ = 0.68, *p* < 0.0001, Figure S4).

## 4. Discussion

We discovered that people who lack notable serum IgG rise after infection by endemic HCoVs exhibit higher nasal IgA levels at the acute phase of their disease. This pattern was observed on anti-N as well as anti-S IgA. These results suggest that IgA in the nasal mucosa is sometimes quickly produced upon HCoV infection, and such rapid local immunity may play a significant role in the clearance of locally produced respiratory viruses. 

Mucosal IgA is produced by IgA-secreting plasma cells in the lamina propria after such cells are primed with differentiation in mucosa-associated lymphoid tissue by antigens recovered from the epithelial layer [10,23]. Vaccination studies on the poliovirus and IAV revealed that mucosal IgA is more efficiently produced upon localized, as opposed to systemic, antigen (live or inactivated) exposure, such as oral vaccination for the poliovirus or inhalation for IAV [12,13,14]. The production of virus-specific mucosal IgA upon HCoV infection was also observed by Callow and colleagues, where inoculation of volunteers by HCoV-229E virus culture resulted in a sharp increase of mucosal IgA from day 7 to 10 post-infection, before plateauing and slowly declining over time [4]. Most IgG non-responders in our study showed a high initial nasal IgA concentration compared to the responders and a relatively fast decline of nasal IgA, which was quicker than the decline observed by Callow et al. [4]. Other data on mucosal IgA dynamics after infection by endemic HCoVs are lacking, and it is, therefore, not possible to make comparisons with other HCoV studies. There are, however, some reports on the mucosal IgA response after SARS-CoV-2 infection. 

At the start of the COVID-19 pandemic, Fröberg et al. reported that anti-N and anti-S IgA against SARS-CoV-2 could already be detected in the nasal mucosa of unvaccinated individuals on day three after infection confirmation (positive RT-PCR test result) [18]. A further publication by the same research group revealed that natural (wild-type) SARS-CoV-2 infection resulted in higher nasal IgA concentration, as opposed to vaccination, though the ACE-inhibiting capacity of the antibodies was not affected [24]. A higher level of nasal IgA could also be found in SARS-CoV-2-infected asymptomatic subjects, suggesting the possible role of nasal IgA in preventing symptomatic (and severe) disease [25]. Aside from the nasal mucosa, secretory IgA could also be studied in saliva. Virus-specific nasal and saliva antibodies are significantly correlated with each other after SARS-CoV-2 infection [26]. The correlation between SARS-CoV-2-specific antibodies in serum and saliva was also reported by a cohort study of Dutch children, irrespective of SARS-CoV-2 exposure, symptoms, or vaccinations [27]. Of note, saliva antibodies decline earlier than serum antibodies in vaccinated and unvaccinated individuals [26,28]. If the nasal IgA kinetics we studied can be compared to saliva antibody kinetics, the rapid decrease of IgA is a phenomenon also found by us. It needs to be mentioned, though, that the COVID-19 studies mentioned above included subjects with no prior exposure or immunity towards SARS-CoV-2. The situation is different for endemic HCoV infection, where the entire adult population has pre-existing B- and T-cell immune memory. A more apt comparison would be with the breakthrough infection cases by SARS-CoV-2 variant Omicron. Only one study has looked at Omicron infections in SARS-CoV-2-vaccinated individuals, and it found anti-SARS-CoV-2 nasal IgA against both the wild-type and the Omicron variant within 28 days after infection [19,20]. 

There is a limitation in our study that needs mentioning. We could not standardize the amount of NPS material collected during nasopharyngeal swabbing, as NPS samples were collected using a swab that was further submerged in UTM. More (or less) swabbing material could have been collected from some subjects than others, and this could have been reflected in higher (or lower) assay signal values. However, we did see in the responder group that IgA rises corresponded with serum IgG rises. Therefore, the variation due to sampling techniques might be limited.

## 5. Conclusions

In this paper, we report that early and high virus-specific nasal-IgA-recognizing HCoVs are particularly present in people without a virus-specific serum IgG rise. In these people, a fast-acting local immune response seems sufficient to clear the infecting virus.

## Figures and Tables

**Table 1:** Demographics and symptom presentation at enrollment of non-responders and responders.

Demographics ^1^	Non-Responder (*n* = 14)	Responder (*n* = 31)	*p* Value ^2^
Age in years	41 (34–56)	51 (36–57)	0.470
Male sex	5 (36%)	12 (39%)	0.848
Smoking past or present	5 (36%)	13 (42%)	0.753
**Comorbidities**			
Lung ^3^	1 (7%)	4 (13%)	0.569
Heart ^4^	2 (14%)	2 (6%)	0.578
Diabetes	0 (0%)	1 (3%)	0.999
**Symptoms at enrollment (V1)**			
Cough	14 (100%)	31 (100%)	NA ^5^
Phlegm	13 (93%)	23 (74%)	0.236
Breathlessness	7 (50%)	18 (72%)	0.749
Wheezing	7 (50%)	8 (26%)	0.172
Runny nose	13 (93%)	23 (74%)	0.236
Fever	4 (29%)	12 (39%)	0.738
Chest pain	5 (36%)	15 (48%)	0.526
Muscle pain	7 (50%)	14 (45%)	0.999
Disturbed sleep	6 (43%)	2 (68%)	0.188
Headache	10 (71%)	22 (71%)	0.999
Generally feeling unwell	12 (86%)	28 (90%)	0.639
Interference with daily activity	9 (64%)	28 (90%)	0.085
Confusion and disorientation	1 (7%)	2 (6%)	0.999
Diarrhea	3 (21%)	1 (3%)	0.082
**Duration of illness/cough prior to V1-sampling in days**			
Illness	7 (3–7.25)	4 (3–5)	0.146
Cough	6 (3–8.25)	4 (2–7)	0.227

^1^ Demographics are presented as number and percentage for categorical variables (male sex, smoking history, comorbidities, and symptoms present at enrollment) or as median and IQR for continuous variables (age in years and duration of illness/cough prior to first sampling date in days). ^2^ *p* value was calculated with Fisher exact test (2-tailed) for categorical variables, or with the Mann–Whitney test for continuous variables. Significance is defined as *p* < 0.05. ^3^ Lung comorbidities include chronic obstructive pulmonary disease, asthma, or other lung conditions. ^4^ Heart comorbidities include heart failure, ischemic heart disease, or other heart conditions. ^5^ The *p* value could not be generated because all subjects in both groups experienced cough.

## Data Availability

The data on viral Ct value and antibody assay are available in the Supplementary Materials. Other demographic data are available upon request to prevent traceability of the study subjects.

## Supplementary Materials

The following supporting information can be downloaded at https://www.mdpi.com/article/10.3390/vaccines12010090/s1: Table S1: HCoV-matched viral Ct value at enrollment and anti-N-antibody dynamics of the subjects in serum and nasal samples. Table S2: Level and dynamics of anti-N antibodies in both serum and NPS samples between serum IgG responders and non-responders. Table S3: HCoV-matched anti-S-antibody dynamics of the subjects in serum and NPS samples. Table S4: Level and dynamics of anti-S antibodies in both serum and NPS samples between serum IgG responders and non-responders. Figure S1: Possible correlation between viral Ct value at V1 with other variables. Figure S2: The level and dynamics of nasal HCoV anti-S IgA between serum IgG non-responders and responders. Figure S3: Correlation between elapsed days from first illness or cough before the start of the study and the nasal anti-N or nasal anti-S IgA value at V1. Figure S4: The correlation between the dynamics of the nasal anti-S and anti-N IgA.
