# Supplementary material for: Endemic Human Coronavirus-Specific Nasal Immunoglobulin A and Serum Immunoglobulin G Dynamics in Lower Respiratory Tract Infections
Source: Vaccines (Basel). 2024 Jan 17;12(1):90. doi: 10.3390/vaccines12010090 (PMC10820673; doi:10.3390/vaccines12010090)
Supplement: Supplementary file 1 [file vaccines-12-00090-s001.zip › vaccines-2784165-supplementary.pdf]

Supplementary material

Endemic Human Coronavirus-Specific Nasal Immunoglobulin A and Serum Immunoglobulin G Dynamics in Lower Respiratory Tract Infections

Ferdyansyah Sechan [1,2], Katherine Loens [3], Herman Goossens [3], Margareta Ieven [3] and Lia van der Hoek [1,2,*]

[1] Laboratory of Experimental Virology, Department of Medical Microbiology and infection Prevention, Amsterdam University Medical Centers, University of Amsterdam, Meibergdreef 9,
1105 AZ, Amsterdam, The Netherlands; m.f.sechan@amsterdamumc.nl
[2] Amsterdam Institute for Infection and Immunity, 1105 AZ Amsterdam, The Netherlands
[3] Laboratory of Medical Microbiology, Vaccine & Infectious Disease Institute (VAXINFECTIO), University of Antwerp, 2610 Antwerp, Belgium; katherine.loens@uantwerpen.be (K.L.); herman.goossens@uza.be (H.G.); greet.ieven@uza.be (M.I.)
* Correspondence: c.m.vanderhoek@amsterdamumc.nl

This file includes

- Table S1-S4
- Figure S1-S4

Table S1: HCoV-matched viral Ct value at enrollment and anti-N-antibody dynamics of the subjects in serum and nasal samples.

ID	HCoV	Virus Ct value at V1[a]	Serum IgG responder[b]	Fold-change serum anti-N IgG	Serum anti-N IgG, x10^5 RLU[c]		Fold-change nasal anti-N IgA	Nasal anti-N IgA, MFI[d]	
					V1	V2		V1	V2
OC-01	OC43	15.07	1	13.24	133.9	236.4	5.64	31.98	180.47
OC-02	OC43	15.66	1	9.99	14.6	145.4	2.49	39.99	99.75
OC-03	OC43	16.05	1	2.58	18.6	48.1	0.56	334.76	189.00
OC-04	OC43	16.3	1	8.93	13.2	117.5	1.70	193.74	328.70
OC-05	OC43	16.64	0	1.04	26.2	27.2	0.19	495.16	95.93
OC-06	OC43	18.17	1	7.07	14.0	98.7	0.43	114.08	49.07
OC-07	OC43	18.46	0	1.18	12.8	15.1	0.44	204.94	90.42
OC-08	OC43	20.27	1	9.19	14.7	135.5	1.31	37.24	48.96
OC-09	OC43	20.62	0	1.30	9.9	12.9	4.60	96.47	443.81
OC-11	OC43	21.02	0	1.02	11.4	11.5	1.20	101.13	120.96
OC-12	OC43	21.31	1	4.71	27.7	130.6	0.54	124.66	67.35
OC-13	OC43	21.51	0	1.08	14.9	16.1	0.55	162.93	89.96
OC-14	OC43	21.79	1	6.90	24.0	165.5	0.86	277.44	238.59
HK-01	HKU1	NA	0	0.66	149.2	98.3	0.18	672.90	118.49
HK-02	HKU1	NA	0	1.03	78.8	81.4	0.73	210.86	153.32
HK-03	HKU1	NA	1	2.16	27.7	59.9	1.91	55.99	106.71
HK-04	HKU1	NA	0	1.05	93.6	98.6	5.15	60.48	311.21
HK-05	HKU1	NA	0	0.97	72.1	70.0	0.22	166.76	36.47
HK-07	HKU1	NA	0	0.88	22.0	19.3	0.33	165.70	55.39
HK-08	HKU1	NA	0	0.87	35.9	31.3	0.75	162.86	122.49
HK-09	HKU1	NA	0	0.81	50.3	40.7	0.29	249.88	71.25
HK-10	HKU1	NA	1	1.51	37.0	55.7	0.98	52.48	51.44
HK-11	HKU1	NA	0	0.61	75.3	45.8	1.35	809.86	1091.87
HK-12	HKU1	NA	1	1.44	88.6	127.9	1.27	61.98	78.50

Table S1 (Continued).

ID	HCoV	Virus Ct value at V1[a]	Serum IgG responder[b]	Fold-change serum anti-N IgG	Serum anti-N IgG, x10[5] RLU[c]		Fold-change nasal anti-N IgA	Nasal anti-N IgA, MFI[d]	
					V1	V2		V1	V2
NL-01	NL63	29.62	1	1.51	111.9	169.0	2.80	56.75	158.95
NL-02	NL63	29.6	1	1.41	161.1	209.7	1.01	68.48	69.28
NL-03	NL63	28.01	1	3.30	80.9	267.1	1.31	43.50	56.92
NL-05	NL63	25.27	1	3.24	113.8	368.6	5.50	129.86	714.85
NL-06	NL63	24.25	1	1.82	198.2	360.0	6.20	32.20	199.76
NL-07	NL63	23.9	1	2.38	152.4	362.2	21.11	69.97	1477.38
NL-08	NL63	23.85	1	2.82	95.7	269.9	1.73	35.87	61.99
NL-09	NL63	22.19	1	2.56	126.3	323.7	13.71	46.90	643.13
NL-11	NL63	18.05	1	2.42	91.2	220.6	14.69	192.16	2822.22
TT-01	229E	17.16	1	13.75	18.0	246.9	1.58	35.99	56.96
TT-02	229E	18.84	1	5.26	35.7	187.5	13.64	65.27	890.33
TT-03	229E	19.33	1	1.81	206.5	290.7	54.86	54.74	3003.44
TT-04	229E	20.5	1	12.17	32.2	391.7	5.98	83.57	499.64
TT-05	229E	21.71	1	5.23	60.4	315.5	1.42	55.50	78.97
TT-06	229E	22.52	1	2.68	122.4	327.5	5.19	45.17	234.25
TT-07	229E	23.91	1	5.25	36.0	189.3	1.02	47.91	48.99
TT-09	229E	25.39	1	7.03	33.0	232.1	3.86	46.99	181.54
TT-10	229E	25.42	1	6.16	27.3	168.1	1.05	32.74	34.50
TT-11	229E	26.88	0	0.88	36.6	17.7	0.10	866.54	89.80
TT-12	229E	27	1	4.19	213.0	187.7	0.92	31.50	28.98
TT-13	229E	27.75	1	1.77	27.4	114.9	2.69	36.47	97.98

a: Ct value of HCoV-NL63, HCoV-OC43, and HCOV-229E were determined by in-house qPCR from nasal sample taken at V1. The test to determine HCoV-HKU1 infection (RespiFinder Plus) did not give any value.

b: Serum IgG responder was determined as serum anti-N IgG fold-change ≥ 1.40

c: RLU = relative luminescence unit

d: MFI = median fluorescence intensity

Table S2: Level and dynamics of anti-N antibodies in both serum and NPS samples between serum-IgG-responders and -non-responders.

Anti-N antibodies	Non-responder, n = 14		Responder, n = 31		P value[c]
	Median	(IQR)	Median	(IQR)	
Serum anti-N IgG at V1, x10^5 RLU[a]	34	(12.5-57.3)	36	(18.6-114)	0.235
Serum anti-N IgG at V2, x10^5 RLU[a]	286	(14,6-40,4)	189	(126-291)	**<0.0001**
Serum anti-N IgG fold-change	0.94	(0.83-10.5)	3.30	(2.16-1.07)	**<0.0001**
Nasal anti-N IgA at V1, MFI[b]	186	(147-540)	55	(37-84)	**<0.0001**
Nasal anti-N IgA at V2, MFI[b]	107	(85-193)	107	(57-329)	0.875
Nasal anti-N IgA fold-change	0.50	(0.21-1.24)	1.73	(1.02-5.64)	**0.0002**

a: RLU = relative luminescence unit
b: MFI = median fluorescence intensity
c: P value is determined by Mann-Whitney test. Bold values = significant difference (p < 0.05)

Table S3: HCoV-matched anti-S-antibody dynamics of the subjects in serum and NPS samples.

ID	HCoV	Serum IgG responder[a]	Fold-change serum anti-S IgG	Serum anti-S IgG, MFI[b]		Fold-change nasal anti-S IgA	Nasal anti-S IgA, MFI[b]	
				V1	V2		V1	V2
OC-01	OC43	1	106.49	1059	112775	192.41	146	28204
OC-02	OC43	1	233.29	307	71621	49.96	672	33616
OC-03	OC43	1	3.40	106	362	1.44	96065	138272
OC-04	OC43	1	165.73	622	103170	49.40	507	25077
OC-05	OC43	0	42.03	935	39293	0.28	711	197
OC-06	OC43	1	2.37	664	1577	1.45	50825	73861
OC-07	OC43	0	5.10	1373	7000	1.33	4636	6187
OC-08	OC43	1	288.19	526	151733	6.23	154	964
OC-09	OC43	0	4.23	84	355	58.12	104	6084
OC-11	OC43	0	9.58	115	1106	68.35	1574	107643
OC-12	OC43	1	3.27	351	1148	0.59	83312	49563
OC-13	OC43	0	514.42	290	149183	2.39	61366	146933
OC-14	OC43	1	2.16	904	1953	9.24	5606	51781
HK-01	HKU1	0	0.13	117617	15017	0.13	4487	574
HK-02	HKU1	0	0.91	942	861	0.13	33083	4261
HK-03	HKU1	1	49.57	344	17053	2.60	64	169
HK-04	HKU1	0	261.57	575	150401	33.27	93	3110
HK-05	HKU1	0	1.53	36	55	0.19	688	131
HK-07	HKU1	0	0.10	14497	1392	0.01	127482	1806
HK-08	HKU1	0	18.82	72	1355	86.35	33	2914
HK-09	HKU1	0	1.39	284	394	0.81	388	313
HK-10	HKU1	1	1534.43	37	56774	1.19	56	67
HK-11	HKU1	0	0.20	144764	29512	1.71	280	478
HK-12	HKU1	1	8.22	176	1447	6.36	541	3442

Table S3 (Continued).

ID	HCoV	Serum IgG responder[a]	Fold-change serum anti-S IgG	Serum anti-S IgG, MFI[b]		Fold-change nasal anti-S IgA	Nasal anti-S IgA, MFI[b]	
				V1	V2		V1	V2
NL-01	NL63	1	1.38	523	721	0.46	1935	888
NL-02	NL63	1	2.32	1010	2344	0.98	369	361
NL-03	NL63	1	5.08	117	594	2.78	270	750
NL-05	NL63	1	6.81	70	477	5.52	595	3289
NL-06	NL63	1	1.57	149	235	3.47	343	1189
NL-07	NL63	1	2.37	71	169	3.94	292	1152
NL-08	NL63	1	1.17	336	392	1.51	308	466
NL-09	NL63	1	3.53	208	737	6.72	110	744
NL-11	NL63	1	3.47	39	136	40.05	452	18128
TT-01	229E	1	10.08	140	1411	6.64	29	195
TT-02	229E	1	167.28	123	20575	23.11	56	1304
TT-03	229E	1	44.21	1686	74562	18.26	418	7646
TT-04	229E	1	261.87	281	73585	66.71	85	5689
TT-05	229E	1	4.08	545	2223	4.56	43	199
TT-06	229E	1	6.67	20785	138652	11.30	641	7247
TT-07	229E	1	2.58	347	896	1.06	381	404
TT-09	229E	1	19.45	147	2869	56.73	148	8412
TT-10	229E	1	4.57	97	443	2.48	31	79
TT-11	229E	0	3.10	1344	4169	0.13	16395	2118
TT-12	229E	1	1.49	138	205	1.00	29	29
TT-13	229E	1	1.62	580	938	1.92	226	435

a: Serum IgG responder was determined as serum anti-N IgG fold-change ≥ 1.40.

b: MFI = median fluorescence intensity

Table S4: Level and dynamics of anti-S antibodies in both serum and NPS samples between serum-IgG-responders and -non-responders.

Anti-S antibodies	Non-responder, n = 14		Responder, n = 31		p value[b]
	median	*(IQR)*	*median*	*(IQR)*	
Serum anti-S IgG at V1, MFI[a]	755	(108-4654)	307	(123-580)	0.192
Serum anti-S IgG at V2 MFI[a]	2781	(745-31958)	1447	(477-56774)	0.744
Serum anti-S IgG fold-change	3.67	(0,73-24,62)	4.57	(2,37-49,57)	0.235
Nasal anti-S IgA at V1, MFI[a]	1143	(237-20567)	309	(85-596)	**0.042**
Nasal anti-S IgA at V2, MFI[a]	2516	(437-2110)	1189	(405-18128)	0.913
Nasal anti-S IgA fold-change	1.07	(0,13-39,48)	4.56	(1,45-18,26)	0.064

a: MFI = median fluorescence intensity.
b: p value determined by Mann-Whitney U test. Significance is defined as $p < 0.05$

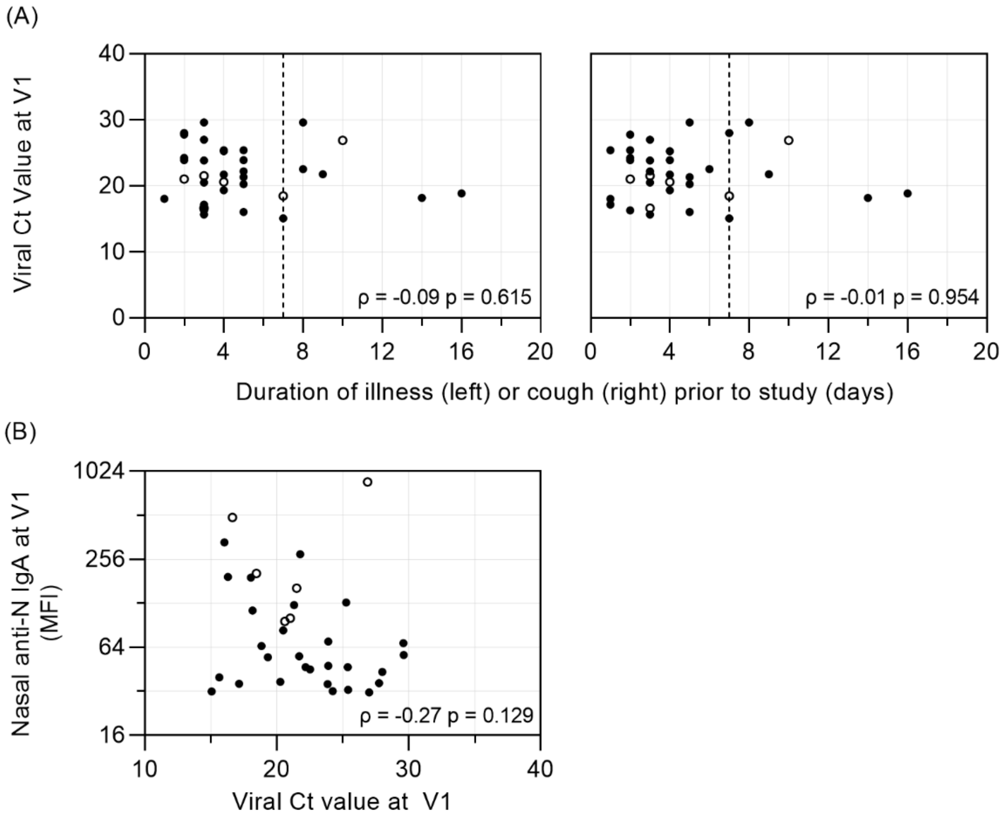

Figure S1: Possible correlation between viral Ct value at V1 with other variables. *(A) The elapsed days from first illness (left panel) or cough (right panel)) to the visit 1(V1) are plotted in x axis (linear scale) against viral Ct value at V1 in y axis (linear scale). Dashed line: duration of illness/cough seven days since first symptom onset. (B) The viral Ct value at V1 presented in x axis is plotted in x axis (linear scale) against the virus-matched nasal anti-N IgA level at V1 in median fluorescence intensity (MFI) in y axis (log 2 scale). Ct values included in this graph is derived from subjects infected by either HCoV-NL63, HCoV-229E, or HCoV-OC43 at V1 (x axis, linear scale) and Black dots: responders. White dots: non-responders. Correlation was evaluated with Spearman's rank correlation test, with p < 0.05 deemed statistically significant.*

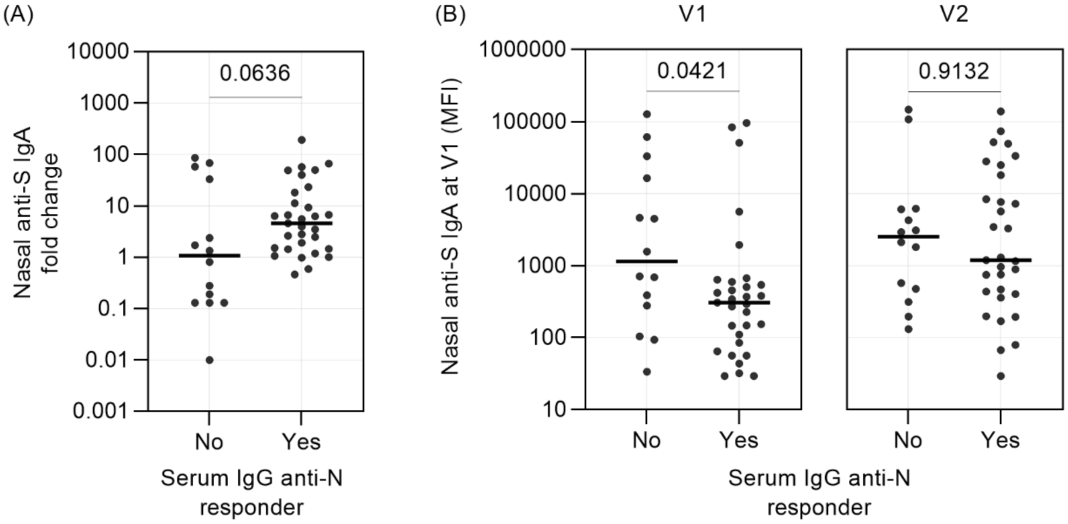

Figure S2: The level and dynamics of nasal HCoV anti-S IgA between serum IgG non-responders and responders. *(A) The fold-change value of nasal anti-S IgA between responders and non-responders. (B) Nasal anti-S IgA level in median fluorescence unit (MFI) at V1 (left panel) and V2 (right panel), grouped between responders and non-responders. Each dot represents one subject and median of each dataset is denoted as a horizontal solid bar. Value distribution between groups was compared with Mann-Whitney test and significance is defined as p < 0.05.*

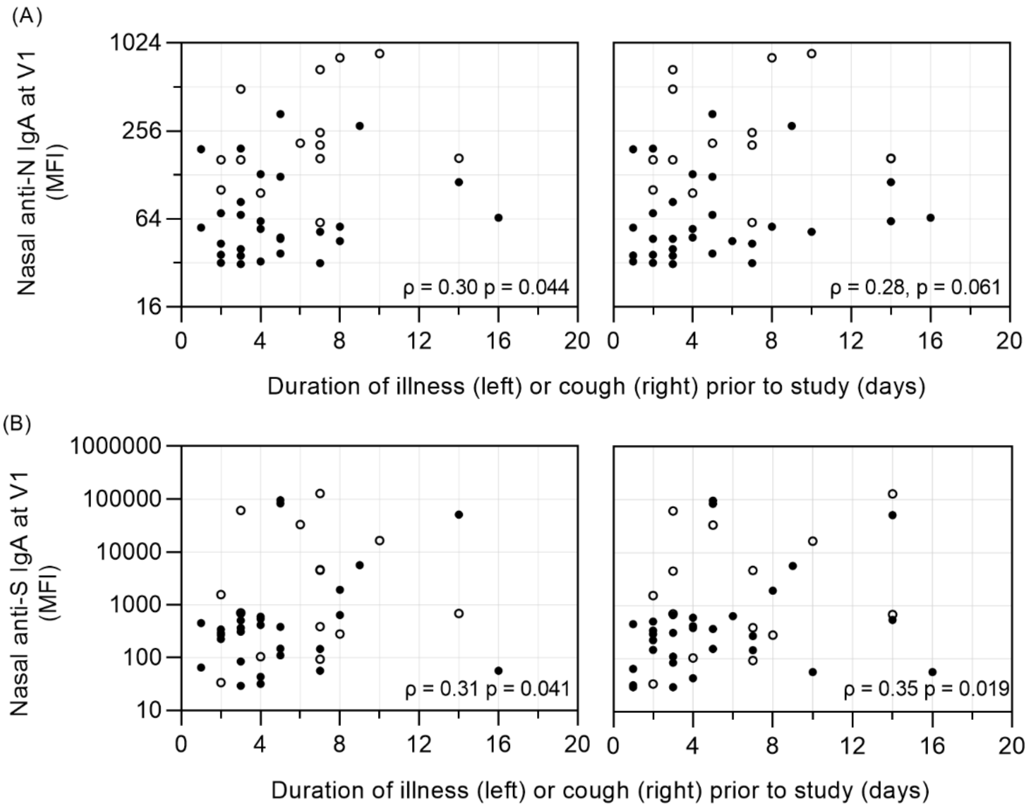

Figure S3: Correlation between elapsed days from first illness or cough before the start of the study and the nasal anti-N or nasal anti-S IgA value at V1. *The elapsed days from first illness (left panel) or cough (right panel)) to the visit 1(V1) are plotted in x axis (linear scale) against nasal anti-N IgA level at V1 (A) or nasal-anti-S IgA level at V1 (B), both in median fluorescence intensity (MFI) in y axis (log 2 scale). White dots: non-responders. Correlation was evaluated with Spearman's rank correlation test, with p < 0.05 deemed statistically significant.*

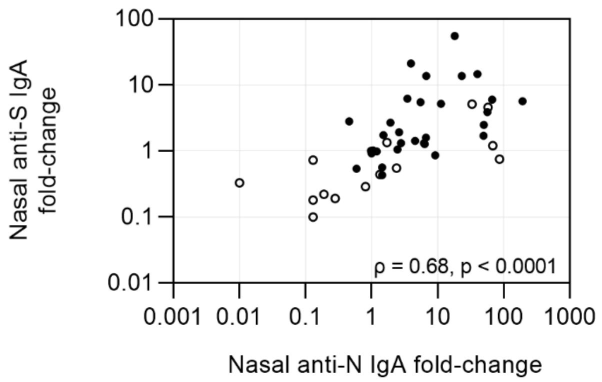

Figure S4: The correlation between the dynamics of the nasal anti-S and anti-N IgA. *The nasal anti-S IgA fold-change was plotted in x axis (log 10 scale) while the nasal-anti-N IgA fold-change was plotted in y axis (log 10 scale). Black dots: responders. White dots: non-responders. Correlation was evaluated with Spearman's rank correlation test, with p < 0.05 deemed statistically significant.*
